# Mechanism of intestinal microbiota disturbance promoting the occurrence and development of esophageal squamous cell carcinoma——based on microbiomics and metabolomics

**DOI:** 10.1186/s12885-024-11982-8

**Published:** 2024-02-22

**Authors:** Xingqiang Huang, Xueyi Chen, Guowei Wan, Dandan Yang, Dongqiang Zhu, Linqian Jia, Jinping Zheng

**Affiliations:** The First Clinical College, Changzhi Medical College, 046000 Shanxi, China

**Keywords:** Esophageal squamous cell carcinoma, Gut microbiota, Metabolomics, Mechanism of carcinogenesis, Cancer

## Abstract

Esophageal squamous cell carcinoma (ESCC) is a high-risk malignant tumor that has been reported in China. Some studies indicate that gut microbiota disorders can affect the occurrence and development of ESCC, but the underlying mechanism remains unclear. In this study, we aimed to explore the possible underlying mechanisms using microbiomics and metabolomics. Fifty ESCC patients and fifty healthy controls were selected as the study subjects according to sex and age, and fecal samples were collected. 16S rDNA sequencing and LC‒MS were used for microbiomics and nontargeted metabolomics analyses. We found significant differences in the composition of the gut microbiota and metabolites between the ESCC patients and control individuals (*P* < 0.05). ESCC patients exhibited increased abundances of Fusobacteriaceae and Lactobacillus, increased levels of GibberellinA34 and decreased levels of 12-hydroxydodecanoic acid; these metabolites could be diagnostic and predictive markers of ESCC. An increase in the abundance of Enterobacteriaceae and Lactobacillus significantly reduced the content of L-aspartate and pantothenic acid, which may be involved in the occurrence and development of ESCC by downregulating the expression of proteins in the pantothenate and coenzyme A biosynthesis pathways. An imbalance in the intestinal flora may decrease the number of eosinophils in peripheral blood, resulting in the activation of an inflammatory response and immune dysfunction, leading to ESCC deterioration. We hypothesize that this imbalance in the gut microbiota can cause an imbalance in intestinal metabolites, which can activate carcinogenic metabolic pathways, affect inflammation and immune function, and play a role in the occurrence and development of ESCC.

**Supplementary Information:**

The online version contains supplementary material available at 10.1186/s12885-024-11982-8.

## Introduction

Esophageal cancer is one of the most aggressive malignant tumors that affects human health. A 2018 global cancer statistics report released by the International Agency for Research on Cancer showed that the global incidence and mortality rates of esophageal cancer were 6.3/100,000 and 5.6/100,000, respectively, and these tumors thus rank seventh and sixth among all malignant tumors in terms of incidence and mortality, respectively [1]. More than 90% of the pathological cases of esophageal cancer in China are esophageal squamous cell carcinoma (ESCC), which has an extremely poor prognosis and a 5-year survival rate of only approximately 20% [2, 3]. The occurrence and development of ESCC are hypothesized to be related to multiple factors, but the specific mechanism underlying ESCC pathogenesis remains unclear. Previous studies have suggested that unhealthy lifestyle habits and an imbalance in the esophageal microbiota may cause the occurrence and development of ESCC [4–6]. The main unhealthy lifestyle risk factors include smoking, drinking and the consumption of hot drinks and nitrosamine, as well as other unhealthy dietary factors [7, 8]. For example, acetaldehyde in alcohol is recognized as a carcinogen, and tobacco also contains a variety of carcinogens, such as nitrosamine, polycyclic aromatic hydrocarbons and active aldehydes. The risk of ESCC increases with an increasing in the intensity and duration of smoking and drinking. Alcohol can promote the absorption of carcinogens by epithelial cells, induce oxidative stress, and promote the transformation of normal esophageal squamous epithelium into tumor cells [9–11]. The consumption of hot drinks and a high-fat diet and a limited intake of fruits and vegetables also increase the risk of ESCC, and the intake of pickled food is associated with an increased risk of death among ESCC patients. For every 25-gram increase in pickled food intake, the risk of death among ESCC patients increases by 6.0% [12–14]. Additionally, long-term smoking, changes in dietary patterns, and chronic inflammation of the esophagus can lead to destruction of the esophageal microenvironment, which significantly increases the risk of Fusobacterium infection. An increase in the occurrence of Fusobacterium infection after esophageal microbiota imbalance is considered a possible cause of ESCC because this process assists in self-colonization of the esophagus and the invasion and occupation of ESCC cells by enriching regulatory T cells and weakening the antitumor immune response [15]. The production of putrescine interferes with polyamine metabolism and increases the expression of substances, such as interleukin (IL)-32/protease 3 (PRTN3), activates multiple signaling pathways, such as the PI3K/AKT pathway and the DNA damage response pathway, and causes ESCC and malignant proliferation [16–18].

The gut microbiota, which is known as the “second genome” of humans, plays a key role in human health and disease. Research shows that gut microbes can activate carcinogenic pathways by affecting the abundance of intestinal metabolites and thus influencing the occurrence and progression of digestive tract tumors [19, 20]. However, to date, most related studies have focused on colorectal cancer, and most studies on bacteria and ESCC have focused on bacteria in the esophagus in situ. However, few studies have investigated the relationship between the gut microbiota and ESCC. Several studies have revealed that the gut microbiota of ESCC patients significantly differs from that of healthy people; compared with those of healthy individuals, ESCC patients exhibit higher levels of proinflammatory and/or carcinogenic bacteria and lower levels of butyric acid-producing and/or anti-inflammatory bacteria [21, 22].

Increased levels of harmful bacteria and decreased levels of beneficial bacteria in the intestine may be involved in the occurrence and development of cancer by causing intestinal metabolic disorder, which may be an undiscovered mechanism involved in the development of ESCC. However, changes in intestinal metabolites in ESCC patients have not been systematically studied, and whether the gut microbiota can affect the occurrence and development of ESCC by changing the abundance of intestinal metabolites has not been confirmed. Therefore, in this study, we performed microbiological and metabolomic analyses to determine the changes in the gut microbiota and intestinal metabolites in ESCC patients and their correlation with the occurrence and development of ESCC. We also provide new evidence for the early diagnosis of ESCC and mechanistic research in this field.

## Materials and methods

### Patient selection

Fifty ESCC patients treated at a hospital in Changzhi city, Shanxi Province, China, from August 2019 to April 2021 were included in the ESCC cohort. Fifty individuals who underwent physical examinations at the same hospital during the same period were selected as the control group in a 1:1 manner based on ethnicity, sex, and age (± 5 years). The inclusion and exclusion criteria for the ESCC group were as follows: (1) had a primary diagnosis of ESCC and agreed to participate in this study; (2) were able to provide pathological diagnosis results, blood and fecal samples, and relevant information; (3) were aged 40 to 80 years; (4) lacked antibiotic use in the 3 months prior to the study; (5) lacked other organ tumors; and (6) had no history of gastrointestinal diseases or gastroesophageal-related surgeries. The inclusion and exclusion criteria for the control group were as follows: (1) agreed to participate in this study; (2) were able to provide blood and fecal samples and relevant information; (3) were aged 40 to 80 years; (4) lacked antibiotic use in the 3 months prior to the study; (5) lacked other organ tumors; and (6) had no history of gastrointestinal diseases or gastroesophageal-related surgeries. All participants or their families provided informed consent for questionnaire completion and sample collection, and the Ethics Committee of Changzhi Medical College approved the study (approval number: RT2021148).

### Collection and processing of fecal samples

A total of 500 mg of fresh feces was collected in a clean test tube. The mixing of urine, water, and other substances during collection was avoided. The samples were stored in a -80 ℃ freezer within 2 h of collection.

The fecal samples for 16S rRNA sequencing were treated as follows: first, 200 mg of fecal sample was weighed, divided into small pieces, added to sterile PBS buffer, and mixed evenly. The mixture was then centrifuged, the supernatant was removed, and the sediment was returned to PBS buffer. The sample was mixed well and centrifuged again to remove the supernatant.

The fecal samples were prepared for liquid chromatography–mass spectrometry (LC‒MS) analysis as follows: the sample was weighed and placed in a 2-mL centrifuge tube, 600 µL of methanol (containing 2-chloro-L- phenylalanine (4 ppm) (stored at -20 °C)) was then added, and vortex oscillation was performed for 30 s. A total of 100 mg of glass beads was added, and the sample was ground in a tissue grinder at 60 Hz for 90 s. Ultrasonication was performed at room temperature for 10 min, after which the sample was centrifuged at 12,000 rpm and 4 ℃ for 10 min. The supernatant was collected and filtered through a 0.22 µM membrane, after which the filtrate was added to the detection bottle.

### DNA extraction

DNA was extracted from all the samples using a Foregene DNA Extraction Kit according to the manufacturer’s instructions. The quantity and quality of the extracted DNA were measured using a NanoDrop NC2000 spectrophotometer (Thermo Fisher Scientific, Waltham, MA, USA) and agarose gel electrophoresis, respectively.

### 16S rRNA gene amplicon sequencing

PCR amplification of the V3–V4 region of the bacterial 16S rRNA gene was performed using the forward primer 338 F (5’-ACTCCTACGGGAGGCAGCA-3’) and the reverse primer 806R (5’-GGACTACHVGGGTWTCTAAT-3’). Sample-specific 7-bp barcodes were incorporated into the primers for multiplex sequencing. The PCR mixture contained 5 µL of buffer (5×), 0.25 µL of FastPfu DNA Polymerase (5 U/µl), 2 µL (2.5 mM) of dNTPs, 1 µL (10 µM) of each forward and reverse primer, 1 µL of DNA template, and 14.75 µL of ddH2O. Thermal cycling consisted of initial denaturation at 98 °C for 5 min; 25 cycles of denaturation at 98 °C for 30 s, annealing at 53 °C for 30 s, and extension at 72 °C for 45 s; and a final extension of 5 min at 72 °C. The PCR amplicons were purified with Vazyme VAHTSTM DNA Clean Beads (Vazyme, Nanjing, China) and quantified using the Quant-iT PicoGreen dsDNA Assay Kit (Invitrogen, Carlsbad, CA, USA). After the individual quantification step, amplicons were pooled in equal amounts, and paired-end 2 × 250-bp sequencing was performed using the Illumina NovaSeq platform with a NovaSeq 6000 SP Reagent Kit (500 cycles) at Shanghai Personal Biotechnology Co., Ltd. (Shanghai, China).

### LC‒MS analysis

#### Liquid chromatography conditions

LC analysis was performed using a Vanquish Ultra-High-Performance Liquid Chromatography (UHPLC) System (Thermo Fisher Scientific, USA). Chromatography was conducted with an ACQUITY UPLC ® HSS T3 (150 × 2.1 mm, 1.8 μm) (Waters, Milford, MA, USA). The column was maintained at 40 °C. The flow rate and injection volume were set to 0.25 mL/min and 2 µL, respectively. For LC electrospray ionization (ESI) (+) MS analysis, the mobile phases were (C) 0.1% formic acid in acetonitrile (v/v) and (D) 0.1% formic acid in water (v/v). Separation was performed using the following gradient: 0 ~ 1 min, 2% C; 1 ~ 9 min, 2%~50% C; 9 ~ 12 min, 50%~98% C; 12 ~ 13.5 min, 98% C; 13.5 ~ 14 min, 98%~2% C; and 14 ~ 20 min, 2% C. For LC ESI (-) MS analysis, the analytes were removed with (A) acetonitrile and (B) ammonium formate (5 mM). Separation was performed using the following gradient: 0 ~ 1 min, 2% A; 1 ~ 9 min, 2%~50% A; 9 ~ 12 min, 50%~98% A; 12 ~ 13.5 min, 98% A; 13.5 ~ 14 min, 98%~2% A; and 14 ~ 17 min, 2% A.

#### Mass spectrum conditions

Mass spectrometric detection of metabolites was performed using a Q Exactive HF-X (Thermo Fisher Scientific, USA) with an ESI source. Simultaneous MS1 and MS/MS (full MS-ddMS2 mode, data-dependent MS/MS) acquisition was also conducted. The parameters were set as follows: sheath gas pressure, 30 arb; aux gas flow, 10 arb; spray voltage, 3.50 kV and − 2.50 kV for ESI(+) and ESI(-), respectively; capillary temperature, 325℃; MS1 range, m/z 81-1000; MS1 resolving power, 60,000 FWHM; number of data-dependent scans per cycle, 8; MS/MS resolving power, 15,000 FWHM; normalized collision energy, 30%; and dynamic exclusion time, automatic.

### Peripheral blood collection and testing

Peripheral blood (2–5 mL) was collected through puncture in labeled EDTA anticoagulant tubes. The white blood cell, red blood cell, hemoglobin, platelet, neutrophil, lymphocyte, monocyte, eosinophil, and basophil levels and the albumin concentration in the peripheral blood were measured using the reactance resistance method, flow cytometry, the bromocresol green method, colorimetry and other techniques.

### Statistical analysis

The results from the epidemiological questionnaire survey and clinical tests were compiled into a database, and statistical analyses were conducted using SPSS 22.0 software. Continuous variables were analyzed using paired t tests, and categorical variables were analyzed using paired chi-squared tests.

The alpha diversity of each sample was evaluated using the Kruskal‒Wallis test and Dunn’s post hoc test, and the rationality of the sequencing depth was reflected by sparse curves. Nonmetric multidimensional scaling (NMDS) analysis and analysis of similarities (ANOSIM) intergroup difference tests were used to measure the differences in the beta diversity of the different groups. Linear discriminant analysis (LDA) effect size (LEfSe) was performed to identify the markers of different groups of gut microbiota. Using the 16S rRNA sequencing results and PICRUSt2 and STAMP software, we predicted the function of microbial metabolites in the samples and identified differentially activated pathways.

The original partial least squares discriminant analysis (OPLS-DA) dimension reduction analysis was performed using the R software package ROPLS with the sample data, and an S-plot diagram was drawn to show the differences in the composition of metabolites among the samples. A permutation test was used to assess the overfitting of the model. R2X and R2Y represent the explanatory power of the established model for the X and Y matrices, respectively. Q2 indicates the predictive ability of the model; the closer the values are to 1, the better the fit of the model, and the more accurately the training set samples can be divided into the original categories.

The *P* value was calculated using statistical tests, the variable importance for the projection (VIP) and fold change were calculated using the OPLS-DA dimension reduction method; the differences in multiple components were evaluated, the strength of the influence and interpretation ability of the levels of each metabolite on sample classification and discrimination were assessed, and marker metabolite screening was performed. If the *P* value was < 0.05 and the VIP value was > 1, the differences in the levels of metabolites were considered statistically significant. For pathway analysis, the MetaboAnalyst software package was used for functional pathway enrichment and topology analyses of the differentially abundant metabolites. The enriched pathways were visualized using the Kyoto Encyclopedia of Genes and Genomes (KEGG) Mapper to analyze the differentially abundant metabolites and pathway maps.

A receiver operating characteristic (ROC) curve was used to predict ESCC diagnosis using the levels of microbes and metabolites, and Spearman correlation analysis was used for the correlation analyses, in which *P* < 0.05 indicated a statistically significant difference.

## Results

### General information

All the patients in the case group and individuals in the control group were Han people from Changzhi City, Shanxi Province, China. A total of 100 stool samples were collected from 50 ESCC patients and 50 control patients. No significant differences in age, smoking or drinking habits, or eating habits, among other characteristics, were found between the ESCC group and the control group (*P* > 0.05), as revealed by paired t tests and paired chi-squared tests; thus, the groups were reasonably matched (Table 1).

**Table 1:** Demographic characteristics of the ESCC and control groups

Factor	ESCC n (%)	Control n (%)	Statistic	*P* value
**Age (years)**	61.91 ± 5.505	61.86 ± 6.456	t = 0.438	0.663
**Sex**				
Male	34 (68.0%)	34 (68.0%)	χ^2^ = 0.033	1.000
Female	16 (32.0%)	16 (32.0%)		
**BMI**				
≤ 24	38 (76%)	21 (42%)	χ^2^ = 0.306	0.760
> 24	12 (24%)	29 (58%)		
**Monthly household income**				
≤ 4000	28 (56.0%)	29 (58.0%)	χ^2^=-0.068	0.946
>4000	22 (44.0%)	21 (42.0%)		
**Drinking habits**				
Yes	16 (32.0%)	16 (32.0%)	χ^2^=-0.033	1.000
No	34 (68.0%)	34 (68.0%)		
**Smoking habits**				
Yes	24 (57.1%)	18 (42.9%)	χ^2^=-0.003	0.271
No	26 (44.8%)	32 (55.2%)		
**Consumption of hot drinks**				
Yes	20 (40.0%)	17 (34.0%)	χ^2^=-0.016	0.617
No	30 (60.0%)	33 (66.0%)		
**Consumption of vegetables and fruits**				
Yes	45 (90.0%)	46 (92.0%)	χ^2^=-0.202	0.920
No	5 (10.0%)	4 (8.0%)		
**Consumption of pickled food**				
Yes	7 (14.0%)	1 (2.0%)	χ^2^=-0.290	0.271
No	43 (86.0%)	49 (98.0%)		

### Species composition and distribution of the gut microbiota

**Fig. 1:**
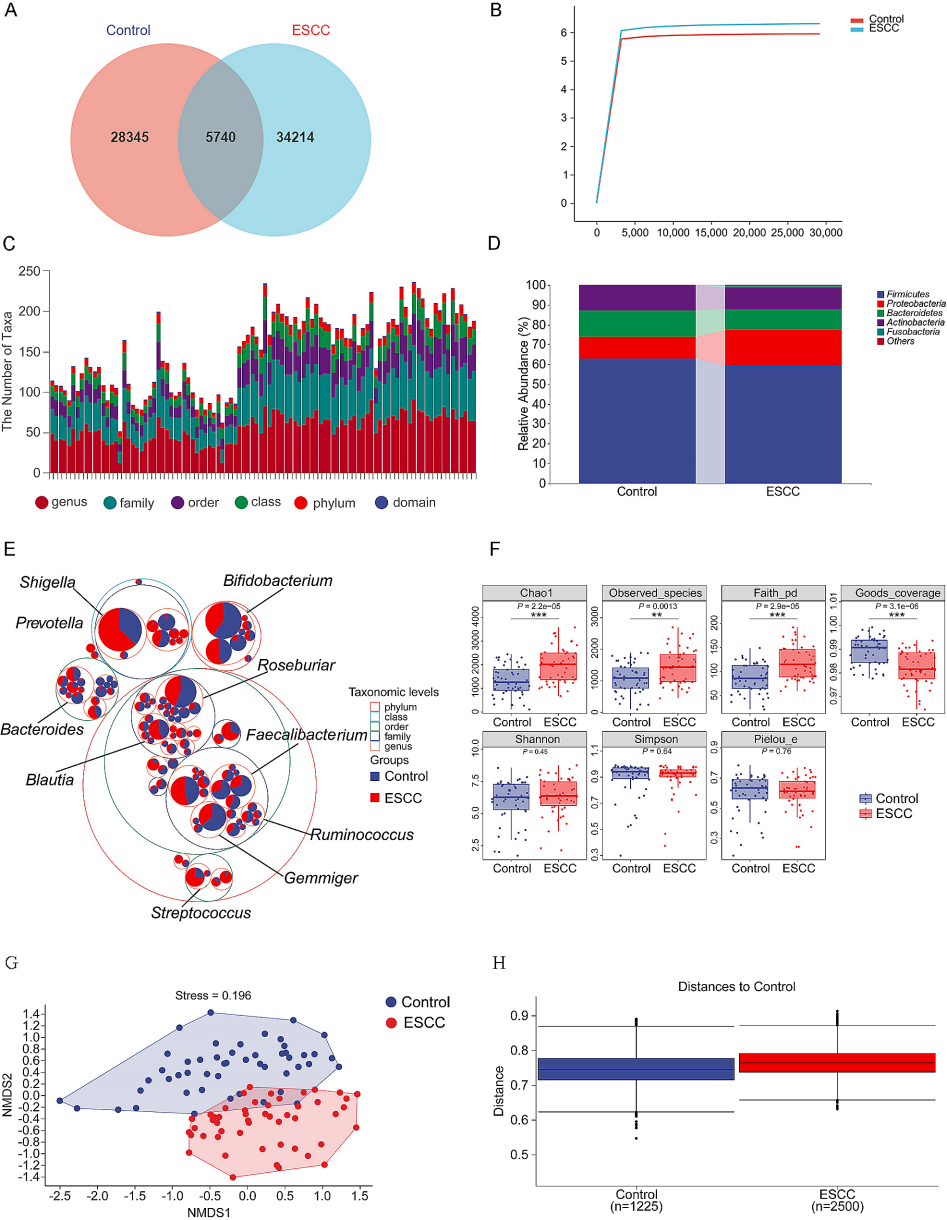
Species composition and diversity analysis. (**A**) In the Wayne chart, the numbers represent the number of OTUs. (**B**) In the sparse curve, the abscissa is the leveling depth, and the ordinate is the median value of the alpha diversity index calculated 10 times. (**C**) The taxonomy of species is shown; the abscissa is the sample, and the ordinate is the number of OTUs at different taxonomic levels. (**D**) The taxonomy composition is shown; the abscissa is the grouping, and the ordinate is the relative abundance of microbes at the phylum level. (**E**) The classification hierarchy tree is shown; the largest circle represents the phylum level, and the circles of gradually decreasing sizes represent the classes, orders, families, and genera. The larger the sector area is, the greater the abundance of the classification unit in the corresponding group. (**F**) For the alpha diversity index, the abscissa is the grouping, and the ordinate is the value of the alpha diversity index. The number under the diversity index label is the *P* value obtained from the Kruskal‒Wallis test, and * indicates significance, as determined by the post hoc Dunn’s test. (**G**) For NMDS analysis, the dots represent samples, and the colors represent the grouping. (**H**) For intergroup difference analysis, the box plot shows the distribution of the distances between samples within a group and a box plot shows the distances between the samples in the groups

The high-quality OTUs obtained after denoising were plotted in a Wayne plot, and the results revealed 34,214 unique OTUs in the ESCC group, 28,345 unique OTUs in the control group, and 5740 OTUs shared by both groups (Fig. 1A). By generating a dilution curve, we found that the curve did not continue to increase with increasing sequencing depth, indicating that the sequencing results covered most of the OTUs of microorganisms in the sample and that our sample size was sufficient to support the findings of this study (Fig. 1B).

The annotation of species taxonomy showed that the microbes in the ESCC group had an average of 1 domain, 11.4 phyla, 21.98 classes, 33.06 orders, 57.18 families, and 69.14 genera. The microbes in the control group had an average of 1 domain, 7.0 phyla, 13.36 classes, 17.66 orders, 30.84 families, and 43.60 genera (Fig. 1C). At the phylum level, the two groups mainly harbored Firmicutes, Proteobacteria, Bacteroidetes, Actinobacteria, and Fusobacteria; Firmicutes was the most important phylum, accounting for 59.03% and 62.84% of the microbes in the ESCC and control groups, respectively (Fig. 1D). At the genus level, although the identities of the dominant species in the two groups were the same, their proportions were different, and the abundances of Shigella, Streptococcus, and Blautia were greater in the ESCC group. In addition, the abundances of Bifidobacterium, Bacteroides, Roseburia, Faecalibacterium, Gemmiger, Prevotella, and Coprococcus were relatively low in the ESCC group (Fig. 1E).

### Analysis of the diversity of the gut microbiota

We measured different alpha diversity indices to evaluate the difference in gut microbiota diversity between the two groups. The richness of the gut microbiota was evaluated using the Chao1 index and Observed_Specifications index. Pielou’s evenness index was used for uniformity evaluation. The diversity was assessed using the Shannon and Simpson indices, and the genetic diversity was assessed using the Faith_ PD index. Species coverage was evaluated using the Goods coverage index, and the Kruskal‒Wallis rank sum test and Dunn’s test were used as post hoc tests to verify the significance of the differences.

Alpha diversity analysis revealed that the Chao1 index, the Observed_Specifications index and the Faith_PD index were greater in the ESCC group than in the control group, and Good’s coverage index was lower in the ESCC group than in the control group; both of these differences were significant. In contrast, no statistically significant differences in the Shannon, Simpson, or Pielou evenness indices were found between the two groups (Fig. 1F). In the beta diversity analysis, an NMDS analysis based on the unweighted UniFrac distance was used to display the community differences between two groups of samples, and the intergroup differences in the NMDS results were tested by ANOSIM. The beta diversity analysis showed obvious separation between the gut microbiota of the two groups (Fig. 1G), and ANOSIM revealed a significant difference between the two groups of samples (*P* < 0.05) (Fig. 1H).

### Identification and functional prediction of marker microbes

**Fig. 2:**
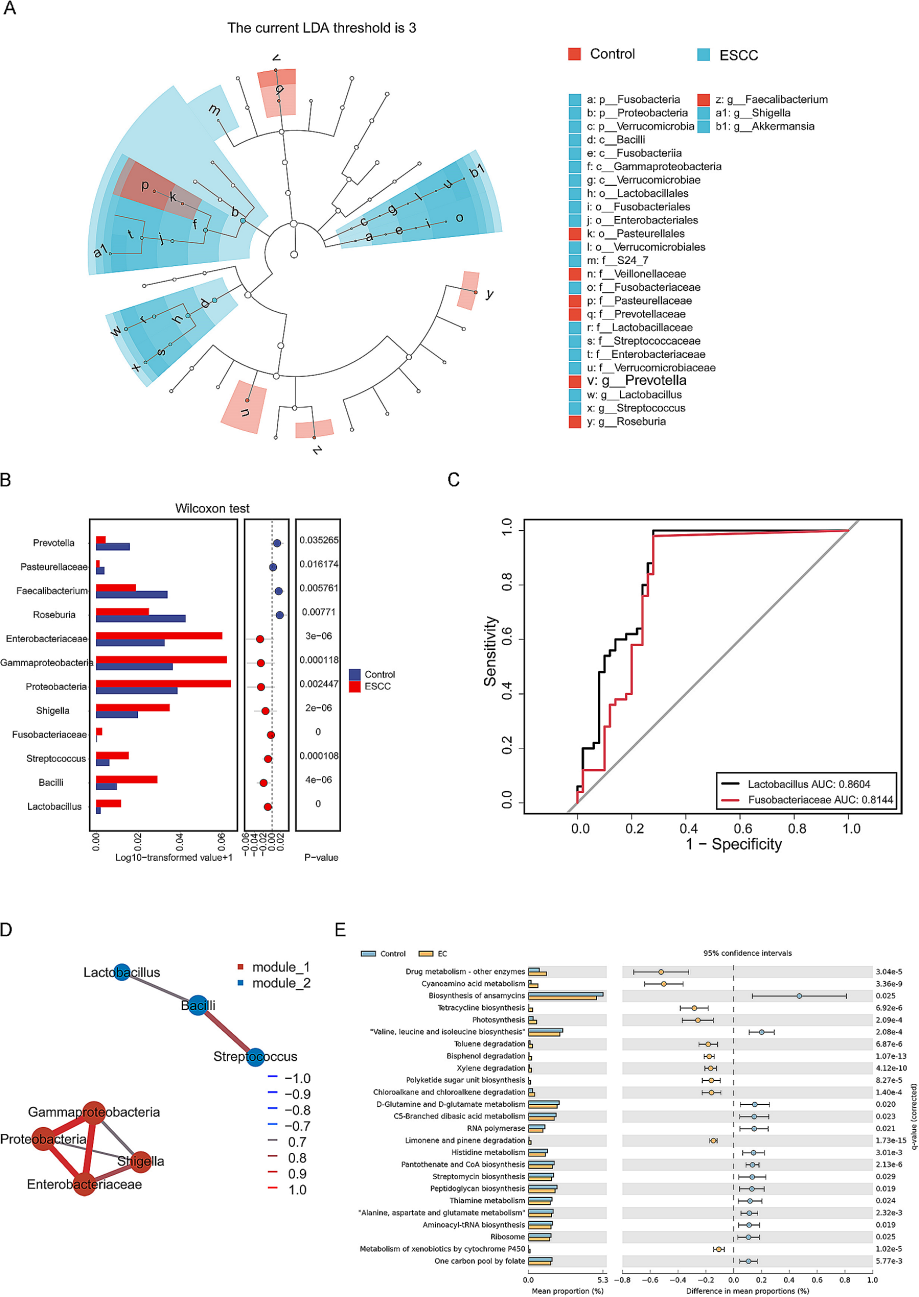
Intestinal marker microbes and function. (**A**) For LEfSe, the vertical axis represents the classification units with significant differences between groups, and the horizontal axis represents the logarithmic scores of LDA for each classification unit. (H) For correlation network analysis, the nodes represent the genera of fungi, and their size is positively proportional to their abundance. A connection between nodes indicates a correlation between two connected nodes. (**B**) In the receiver operating characteristic curve, the abscissa represents 1-specificity, and the ordinate represents specificity. (**C**) The relative abundance of bacterial communities was compared; the horizontal axis represents the grouping, and the vertical axis represents the relative abundance. (**D**) Correlation network analysis was performed. (**E**) Pathway enrichment analysis was performed; the left column shows the abundance distribution of metabolic pathways (percentage of all metabolic pathways) in the two groups, and the right column shows the *P* value of the difference between groups

LEfSe was used to identify specific differentially abundant gut microbes in the ESCC cohort. The LDA histogram showed that 28 marker microbes were differentially expressed between the two groups with an LDA threshold = 3 (Fig. 2A). The microbes with a significantly lower abundance in the ESCC group than in the control group included Pasteurellaceae and Pasteurellales, as well as Roseburia, Faecalibacterium, Veillonellaceae, Prevotellaceae and Prevotella within Pseudomonadota. The microbes with a significantly greater abundance in the ESCC group than in the control group included Proteobacteria, Gammaproteobacteria and Enterobacteriaceae within Pseudomonadota; Shigella, Streptococcus and Lactobacillus within Bacillota; Bacillus, Verrucomicrobiaceae and Akkermansia within Verrucomicrobiota; and Fusobacteriaceae (Fig. 2B).

By plotting the ROC curve and calculating the area under the curve, the potential value of the marker microbes in diagnosing ESCC was explored. The results showed that the areas under the curve generated using the levels of Fusobacteriaceae and Lactobacillus were 81.44% and 86.04%, respectively, indicating a significant increase in the abundance of these microbes in the gut of the ESCC group; these microbes thus distinguished the ESCC group from the control group (Fig. 2C).

Subsequently, an association network analysis based on the Spearman correlation coefficient was conducted to explore the relationships between the marker bacterial communities discovered by LEfSe. With the screening conditions *P* value < 0.05 and Q value < 0.05, we found that Streptococcus, Lactobacillus and Bacillus, which belong to Bacillota, were in the same module and that the Bacillus levels were positively correlated with the Streptococcus and Lactobacillus levels; in addition, Proteobacteria, Gammaproteobacteria, Enterobacteriaceae and Shigella, which belong to Pseudomonadota, were localized in another module, and their levels were positively correlated with each other (Fig. 2D).

PICRUSt2 software was used to investigate the phylogeny of the microbes in the ESCC and control groups, and STAMP software was used to analyze the differences in the results to predict the potential function of the gut microbiota. We found a total of 25 significantly enriched KEGG metabolic pathways between the two groups (Fig. 2E); among these pathways, 11 metabolic pathways were significantly activated in the ESCC group, and these included drug metabolism-other enzymes, cyanoamino acid metabolism, tetracycline biosynthesis, photosynthesis, toluene degradation, bisphenol degradation, xylene degradation, polyketide sugar unit biosynthesis, chloroalkane and chloroalkane degradation, limonene and pinene degradation, and the metabolism of xenobiotics by cytochrome P450.

Fourteen metabolic pathways were significantly inhibited in the ESCC group, and most of the inhibited pathways were associated with amino acid metabolism. The fourteen inhibited pathways were as follows: the biosynthesis of ansamycins; the degradation of valine, leucine and isoleucine; D-arginine and D-ornithine metabolism; C5 branched basic acid metabolism; RNA polymerase; histidine metabolism; pantothenate and CoA biosynthesis; streptomycin biosynthesis; peptidoglycan biosynthesis; thiamine metabolism; alanine, aspartate and glutamate metabolism; aminoacyl-tRNA biosynthesis; ribosomes; and one-carbon pool containing folate.

### Analysis of differentially abundant metabolites in the intestine

**Fig. 3:**
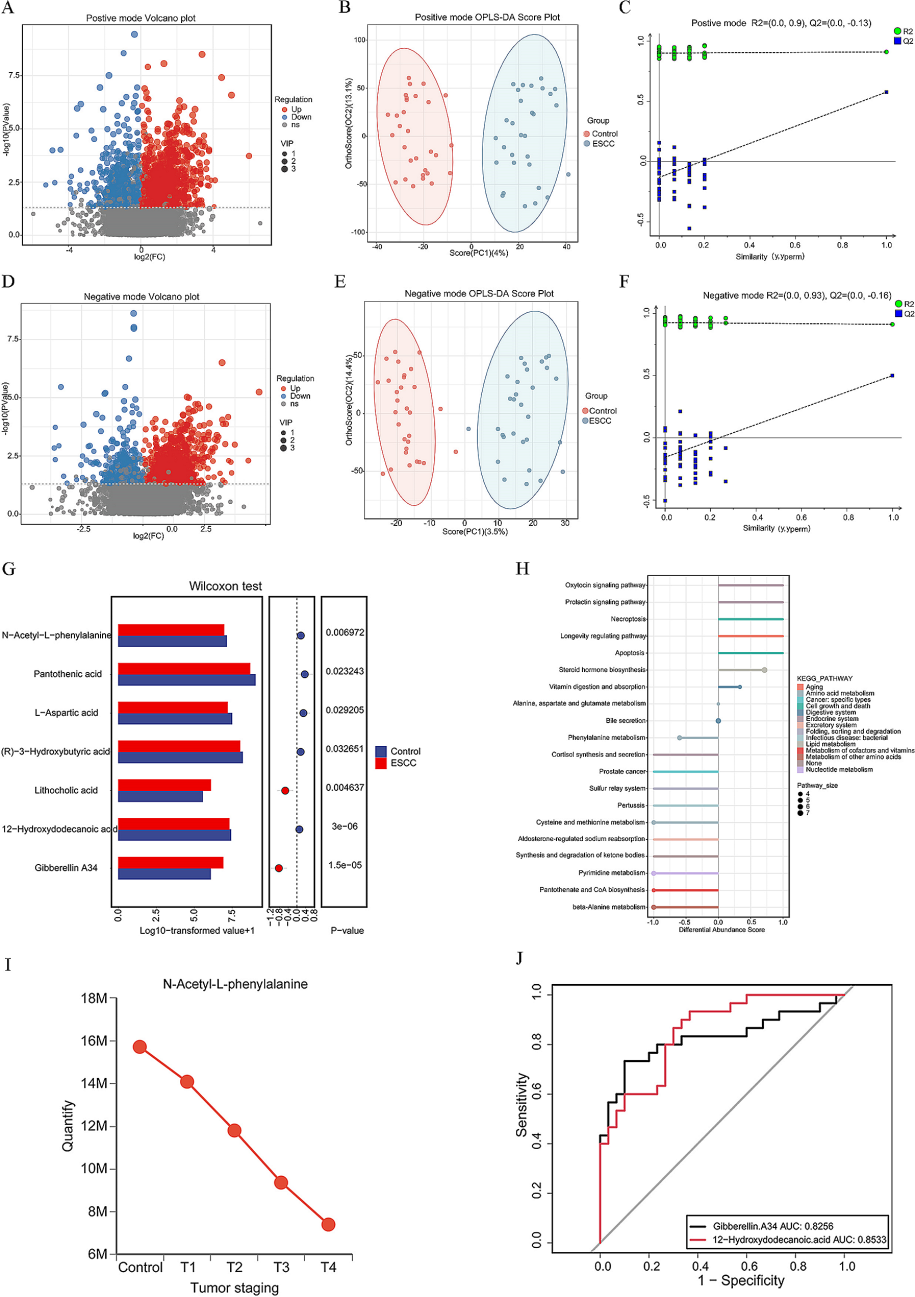
Differentially abundant metabolites and functions in the intestine. (**A**) (**D**) A volcano plot of the positive and negative ion modes is shown. The abscissa represents the logarithm (log2) value, which represents the quantitative difference in multiple metabolites between the two samples; the y-axis represents the -log10 of the *P* value. (**B**) (**E**) An OPLS-DA score map in the positive and negative ion modes is shown; the abscissa represents the decomposition degree of the first main component, and the ordinate represents the decomposition degree of the second main component. (**C**) (**F**) The OPLS-DA permutation test diagram in the positive and negative ion modes is shown. If this diagram meets any of the following criteria, the results are reliable and effective: (1) the intersection between the regression line and the ordinate of the point is less than 0, and (2) all blue Q2 points are lower than the original blue Q2 point on the far right. (**G**) In the multifunctional difference test chart, the left bar chart represents the mean level of each product in different groups, the middle chart shows the 95% confidence interval with an error line, and the right chart shows the *P* value of the difference in the levels of the product between the two groups. (**H**) In the differential enrichment score plot, the horizontal axis represents the DA score, where DA score = (number of substances with increased levels - number of substances with decreased levels)/total number of differentially regulated substances in the pathway. The vertical axis represents the metabolic pathway, and the columns on the left and right represent the pathways inhibited and activated, respectively, in the ESCC group. The size of the top column represents the number of enriched differentially abundant metabolites in the pathway. (**I**) A line chart of the N-acetyl-L-phenylalanine content is shown; the abscissa is the tumor stage, and the ordinate is the content. (**J**) The receiver operating characteristic curve is shown; the abscissa represents 1-specificity, and the ordinate represents specificity

Several studies have shown that *Helicobacter pylori* infection may have an impact on the gut microbiota. To prevent this infection from influencing this experiment as much as possible, we selected 30 fecal samples from the ESCC group and the control group with or without *H. pylori* infection as matching conditions for the LC‒MS-based nontargeted metabolomic analysis of feces. In positive ion mode, 2577 differentially abundant metabolites were found among the primary metabolites of the two groups (1606 with higher levels in the ESCC group and 971 with lower levels in the ESCC group) (Fig. 3A). In negative ion mode, 1404 differentially abundant metabolites were found among the primary metabolites of the two groups (955 with higher levels in the ESCC group and 449 with lower levels in the ESCC group) (Fig. 3D). The OPLS-DA results showed that the levels of metabolites in the two groups were significantly different (Fig. 3B and E), and the permutation test showed that the OPLS-DA results were reliable and effective (Fig. 3C and F). Using a *P* value < 0.05 and VIP > 1.0 as the screening criteria, a total of 168 differentially abundant metabolites were found in the first-level substance list of the samples. Among these, 88 metabolites had significantly higher levels in the ESCC group than in the control group, and the majority of these metabolites were classified as steroids and their derivatives, fatty acids, and conjugates. The content of 80 metabolites was significantly lower in the ESCC group than in the control group, and the majority of the metabolites were classified as amino acids, peptides, or analogs (Supplementary Figure 1).

The metabolite with the most significant increase among the secondary differentially abundant metabolites in the ESCC group was gibberellin A34, whereas the metabolite with the most significant decrease was 12-hydroxydodecanoic acid. In addition, we found that the levels of secondary bile acid in the ESCC group were significantly increased. The levels of L-aspartic acid, pantothenic acid, and the butyrate metabolite (R)-3-hydroxybutyric acid were significantly decreased in the ESCC group (Fig. 3G).

The MetPA database was used to analyze the metabolic pathways related to the differentially abundant metabolites between the ESCC patients and control individuals. Hypergeometric tests were used for the data analysis, and the topological structure of the metabolic pathways was characterized by relative centrality. A total of 168 differentially abundant metabolites were mapped to 75 different KEGG metabolic pathways. Among the top 20 metabolic pathways according to importance, 7 metabolic pathways in the ESCC cohort were significantly activated, including cell growth and death, the endocrine system, lipid metabolism and aging. Eleven metabolic pathways were significantly inhibited, and these pathways included amino acid metabolism in the body. These findings are consistent with the pathway analysis results (Fig. 3H). The impacts of the metabolites on the pathways are detailed in Schedule S1. Thirty ESCC patients underwent T staging according to the 2017 Joint Committee on Cancer Control (JACC) 8th edition tumor staging standards, and changes in metabolite levels during ESCC progression were observed. These patients included 6 T1-stage patients, 6 T2-stage patients, 12 T3-stage patients, and 6 T4-stage patients. We found a significant decrease in the level of N-acetyl-L-phenylalanine in the ESCC group. Although one-way ANOVA did not reveal significant differences in the levels of this metabolite among patients with different stages of ESCC, a gradual decrease in the level of N-acetyl-L-phenylalanine was observed during ESCC progression (Fig. 3I). To explore the potential value of the identified differentially abundant metabolites in diagnosing ESCC, we generated ROC curves and calculated the areas under the curve (AUCs). The results showed that the AUCs obtained using the gibberellin A34 and 12-hydroxydodecanoic acid levels were 82.56% and 85.33%, respectively, which indicated that these metabolites could distinguish the ESCC group from the control group (Fig. 3J).

### Correlations between the gut microbiota and metabolites

According to the correlation network analysis, microorganisms located in the same module may be more closely related in terms of survival or function. The potential relationship between the gut microbiota and intestinal metabolites was further explored. In this subsequent analysis, Proteobacteria, Gammaproteobacteria, Enterobacteriaceae and Shigella in Module 1 and Streptococcus, Lactobacillus and Bacillus in Module 2, which were identified from the association network, were used to construct a Spearman rank correlation coefficient thermogram with the differentially abundant metabolites that had a significant impact on the top 20 metabolic pathways (screening conditions: *P* value < 0.05, R value = 0.25).

**Fig. 4:**
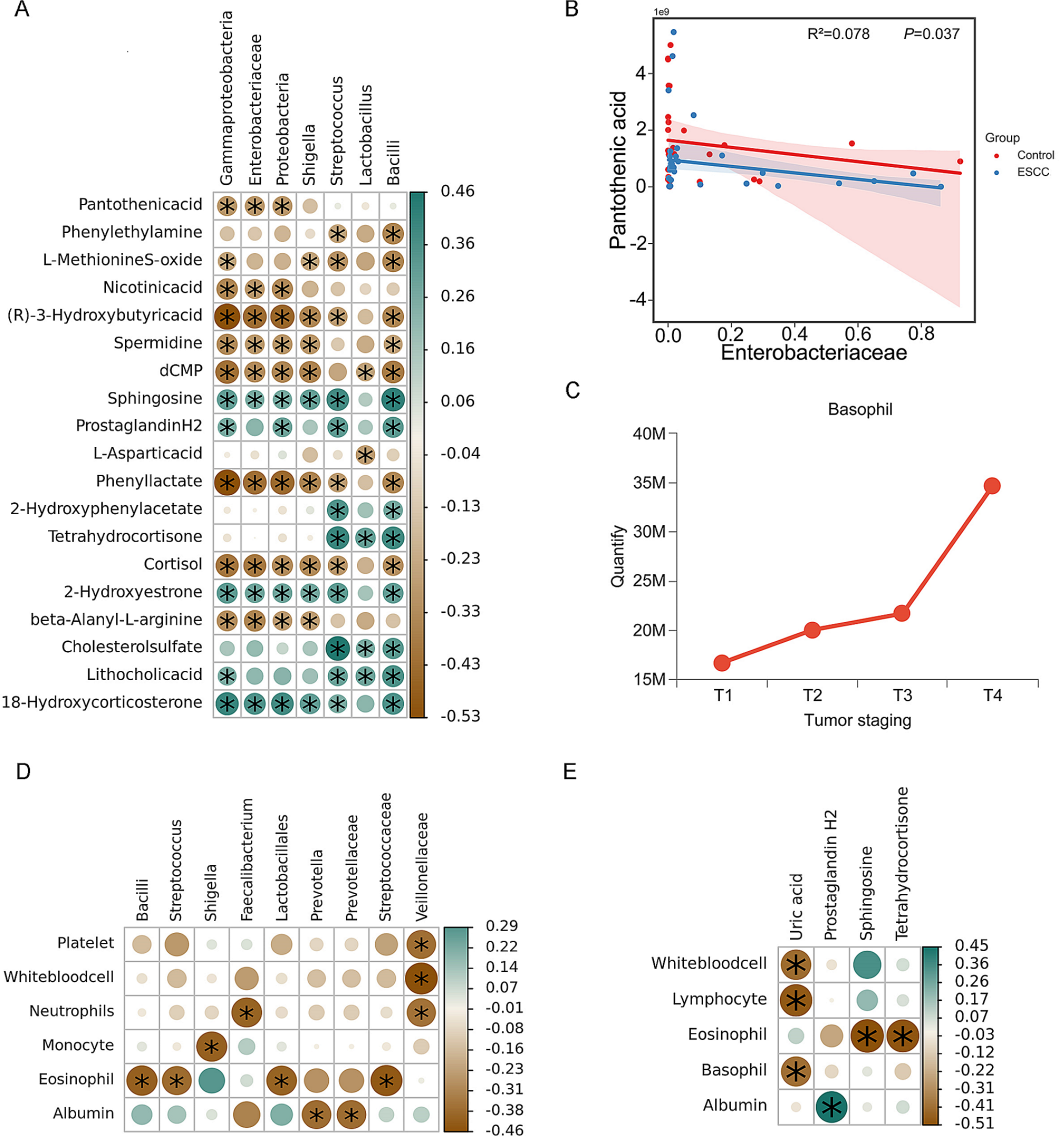
Correlation analysis. (**A**) A thermogram of the correlation coefficient between the gut microbiota and intestinal metabolites is shown; blue represents a positive correlation, green represents a negative correlation, the color depth represents a strong or weak correlation, * indicates *p* < 0.05, and the number on the right represents the correlation coefficient. (**B**) For the line chart of the number of basophils in the peripheral blood, the abscissa is the tumor stage, and the ordinate is the number of cells. (**C**) In the linear relationship diagram, the X axis is the independent variable (gut microbiota), and the Y axis is the dependent variable (pantothenic acid). (**D**) A thermogram of the correlation coefficient between the gut microbiota and the number of peripheral blood cells is shown. (**E**) A heatmap of the correlation coefficients between intestinal metabolites and the number of peripheral blood cells is shown

We found a negative correlation between the bacterial community of Module 1 and pantothenic acid and (R)-3-hydroxybutyric acid, and the bacterial community in Module 2 was positively correlated with lithocholic acid (Fig. 4A). In addition, Streptococcus in the gut microbiota was positively correlated with the intestinal metabolites sphingosine and tetrahydrocortisone, and a negative correlation was found between Lactobacillus and L-aspartic acid. A significant decrease in the L-aspartic acid and pantothenic acid contents in the ESCC group affected the pantothenate and CoA biosynthesis metabolic pathways, which were significantly inhibited in the ESCC group, as revealed by analyzing microbial communities and metabolic product pathways. We further conducted a simple linear regression model analysis of the different microbiota and differentially expressed metabolites and explored the linear relationships between these two variables. The results showed a linear relationship between Enterobacteriaceae abundance and pantothenic acid content (R^2^ = 0.078, *P* = 0.037) (Fig. 4B).

### Relationships among the gut microbiota, metabolites, and changes in the peripheral blood cell count

To explore the changes in the number of blood cells in the peripheral blood after alterations in the gut microbiota and intestinal metabolites, we measured the levels of white blood cells, red blood cells, hemoglobin, platelets, neutrophils, lymphocytes, monocytes, eosinophils, basophils and albumin in the peripheral blood of 30 ESCC patients and analyzed them by single factor variance (Schedule S2). Although univariate ANOVA did not reveal significant differences in the levels of blood indicators among patients with tumors in different stages, the basophil content gradually increased during ESCC progression (Fig. 4C). A Spearman rank correlation coefficient heatmap was generated to determine the correlation between the number of blood cells in the peripheral blood and the microbial community and metabolites. Eosinophil counts were negatively correlated with Streptococcus abundance in the gut microbiota and with the intestinal metabolites sphingosine and tetrahydrocortisone (Fig. 4D and E).

## Discussion

ESCC is the most common type of esophageal cancer in China, and most patients are diagnosed with this disease in the middle to late stages. However, the specific mechanism underlying ESCC pathogenesis remains unclear, and no molecular markers that can be widely applied have been identified. The human intestinal tract consists of a complex microbial environment. The role of the gut microbiota and fecal metabolite disorders in the occurrence, development, diagnosis and treatment of tumors has attracted much attention, but determining the specific associations of these disorders with ESCC will require further research. Therefore, we adopted microbiological and metabolomic methods to explore the changes in the gut microbiota structure and fecal metabolites in ESCC patients, studied the role and mechanism of gut microbiota disturbances in disrupting the intestinal metabolic environment to promote the occurrence and development of ESCC, and provided new ideas for the prevention and treatment of ESCC.

To minimize the impact of diet, lifestyle and other factors in this study, we conducted an epidemiological questionnaire survey and statistical analysis of data from ESCC patients and a control population. The paired chi-square test results showed no significant differences in smoking, drinking, eating habits or other aspects between the two groups (*P* > 0.05), indicating that our matching process was relatively reasonable. The diversity of the gut microbiota is often regarded as an indicator of a healthy gut microbiome, and changes in diversity are related to the deterioration of health or disease. Previous studies have revealed that the alpha diversity of the gut microbiota is not significantly different between ESCC patients and healthy controls, but the beta diversity is significantly different [21]. These findings are basically consistent with the results of our study. The alpha diversity analysis revealed no significant differences in diversity or evenness between the ESCC patients and healthy controls. However, we observed a significant increase in the richness and genetic diversity of ESCC patients, as well as a significant decrease in coverage. An increase in bacterial richness may indicate the excessive growth of harmful bacteria in the patient’s gut. In addition, beta diversity analysis revealed significant differences in the spatial distributions of the two groups of flora, which indicated that the gut microbiota may be strongly affected by the tumor load. The LEfSe results further verified this speculation; the microflora of the ESCC group significantly differed from that of healthy people. Specifically, the ESCC group showed significant increases in the abundance of Shigella, which belongs to the phylum Proteobacteria; Streptococcus, which belongs to the phylum Firmicutes; and other pathogenic bacteria and conditional pathogens in the intestinal tract. There were also significant decreases in the abundance of human intestinal symbiotic bacteria, such as Roseburia, which belongs to the phylum Firmicutes; Prevotella, which belongs to the genus Bacteroidetes; and an unbalanced proportion of Firmicutes and Bacteroides.

An increase in the level of harmful bacteria and a decrease in the level of beneficial bacteria in the intestinal tract have been proven to be related to the occurrence and development of tumors. An increase in several specific groups of harmful bacteria, such as *Escherichia coli* and enterotoxigenic *Bacteroides fragilis*, has been associated with chronic tissue inflammation and the release of proinflammatory and carcinogenic mediators, thus increasing the likelihood of developing colorectal cancer following the inflammation-dysplasia-cancer sequence in inflammatory bowel disease patients [23]. In pancreatic cancer, intestinal microbes may move from the intestinal tract to the pancreas through the intestinal barrier and release cell components, which shut down the immune system or cause inflammation and thus promote tumor growth [22]. In addition, research has shown that chronic inflammation plays a key role in the progression of ESCC, and an imbalance in Bacillota and Bacteroidota abundance in the intestine is associated with the development of intestinal inflammation [24]. At the genus level, Streptococcus was found to be the dominant species in the ESCC group in our study. This pathogenic gram-positive bacterium can produce lactic acid after infection and induce epithelial cells to secrete proinflammatory cytokines. The accumulation of lactic acid plays an important role in processes involved in tumorigenesis, such as angiogenesis, cell migration and metastasis, whereas proinflammatory cytokines can cause immunopathological changes or apoptosis and thus promote tumor progression [25, 26].

In this study, the metabolites whose levels were reduced in the intestinal tract of the ESCC group were mainly amino acids, peptides, and analogs. Pathway analysis of the microbiota and metabolites also revealed significant inhibition of multiple amino acid metabolism pathways in the ESCC group, indicating that there was a significant disruption in amino acid metabolism in these patients, which could possibly affect the occurrence and development of ESCC. Amino acids and their products play important roles in metabolic syndromes, nervous system disease, cancer treatment and prevention and other diseases. In the intestinal tract, amino acids interact with microorganisms to maintain the balance of the internal intestinal environment, and immune cells in the body also rely on amino acid-dependent methods for obtaining energy [27]. During the proliferation and differentiation of tumor cells, amino acids play important regulatory roles as substrates and regulatory factors in many metabolic pathways, including ATP production, nucleotide synthesis, and redox balance, to support the function of malignant cells [28, 29]. The content of intestinal metabolites may vary during different stages of ESCC development. We found that the content of N-acetyl-L-phenylalanine was significantly decreased in the ESCC group, although one-way ANOVA did not reveal significant differences in the level of this metabolite among patients with different stages of ESCC, which may have been related to our sample size being too small. However, we observed a gradual decrease in content during ESCC progression. A decrease in the N-acetyl-L-phenylalanine content mainly inhibits the phenylalanine metabolic pathway. A study of fecal metabolomics has shown that phenylalanine metabolism pathways are significantly enriched in colorectal cancer patients and are associated with the development of colorectal cancer [30]. Another study revealed that phenylalanine can be used as a diagnostic biomarker for metastatic ESCC patients [31]. These findings suggest that N-acetyl-L-phenylalanine may also play an important role in the proliferation and metastasis of ESCC, and a decrease in the intestinal content of N-acetyl-L- phenylalanine may indicate a poor prognosis in ESCC patients.

These results indicate that the gut microbiota and intestinal metabolites of ESCC patients are imbalanced. We further explored the relationships between the two groups by correlation network analysis and Spearman correlation analysis. Microbes in the same module in the correlation network may be more closely related in terms of survival or function. Correlation analysis revealed that differences in the levels of metabolites were related to changes in the abundance of specific species. The presence of abnormally enriched metabolites in ESCC patients was due to either the imbalance of gut microbes or their interactions. The abundances of Proteobacteria, Gammaproteobacteria, Enterobacteriaceae and Shigella were negatively correlated with the level of the butyrate metabolite (R)-3-hydroxybutyric acid in Module 1 of the correlation network, and the abundances of Streptococcus, Lactobacillus and Bacillus in Module 2 were positively correlated with the levels of secondary bile acid and lithocholic acid. Data from mechanistic studies demonstrate the ability of the gut microbiota to interact with the colonic epithelia and immune cells in the host via the release of a diverse range of metabolites, proteins and macromolecules that regulate CRC development [32]. Research shows that bile acid is closely involved in the occurrence of ESCC. An imbalance in the gut microbiota and changes in the fecal bile acid composition may be involved in the regulation of inflammatory reactions through G protein coupling to the bile acid receptor TGR5. Bile acid in the intestine can also directly and rapidly affect the overall metabolism of the gut microbiota through processes including cell membrane damage and amino acid, nucleotide and carbohydrate metabolism disorders [33, 34]. A reduction in the level of the butyrate metabolite (R)-3-hydroxybutyric acid significantly inhibited the biosynthesis and metabolism of ketones. Ketone bodies not only play an important role in energy homeostasis but also have many functions, such as regulating immune cells, affecting the gut microbiota, and inhibiting inflammation, and antiaging β-hydroxybutyric acid can promote the M2-type polarization of macrophages through the transduction and transcription activating factor 6 signaling pathway, thereby alleviating colitis in mice [35]. These results indicate that the metabolic balance of the gut microbiota in ESCC patients is disrupted and may affect the occurrence and development of ESCC by regulating multiple metabolic pathways.

**Fig. 5:**
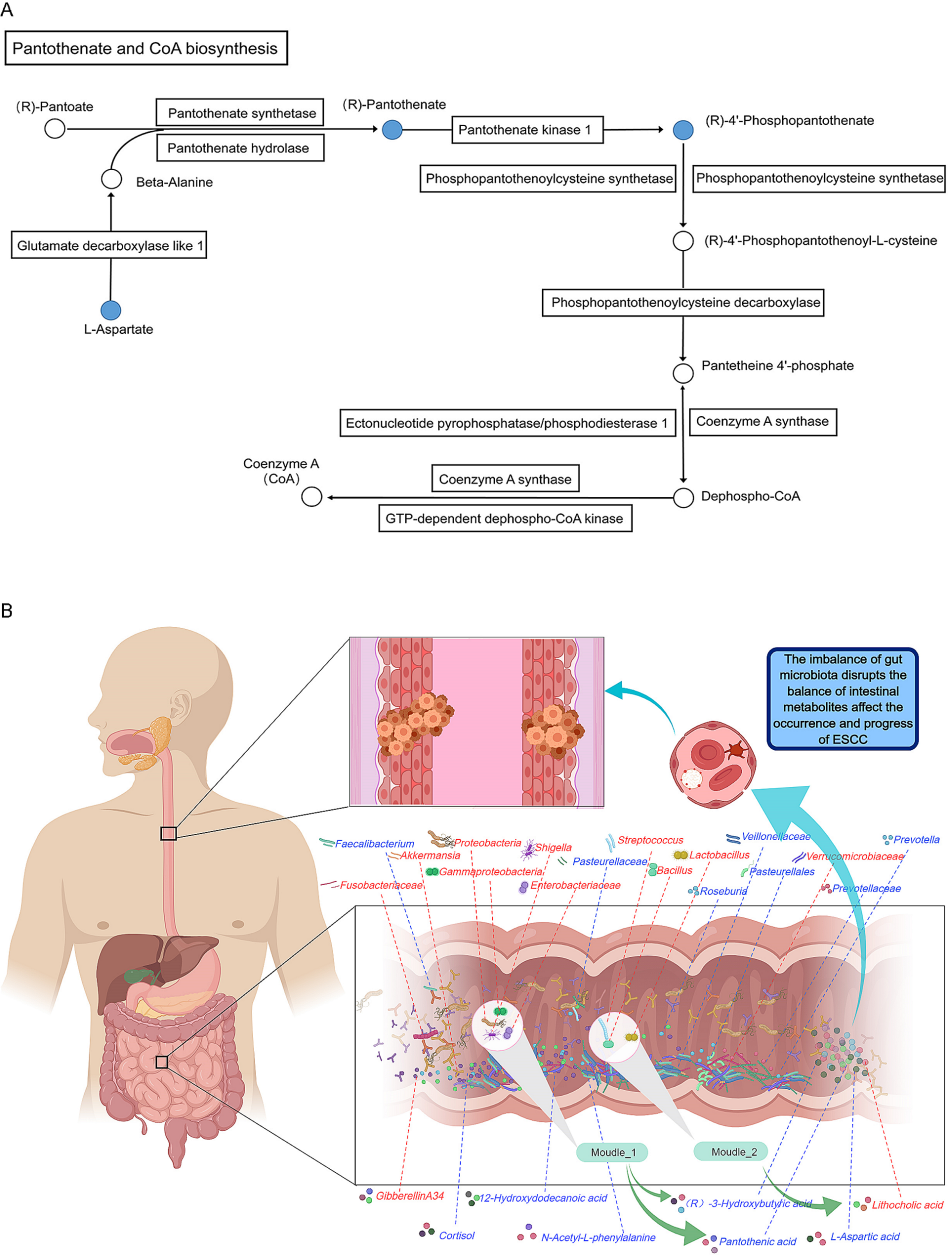
Schematic diagram of the pathogenic mechanism. (**A**) In the pantothenate and CoA biosynthesis pathways, the boxes represent protein molecules, the circles represent metabolic molecules, and the blue color represents decreases in the levels of substances. (**B**) Relationships among the gut microbiota, intestinal metabolites, number of peripheral blood cells and ESCC incidence are depicted; blue indicates a decrease, and red indicates an increase

We found significantly reduced levels of pantothenic acid and L-aspartic acid in the intestinal tract of ESCC patients, which likely affects the biosynthesis pathway of pantothenate and coenzyme A. This pathway was significantly inhibited in the ESCC group, as revealed by microbial and fecal metabolite pathway analysis. Pantothenic acid was negatively correlated with the flora in Module 1 in the correlation network and exhibited a linear relationship with Enterobacteriaceae in the gut microbiota. The L-aspartic acid levels were negatively correlated with the abundance of Lactobacillus. Pantothenic acid and its metabolite coenzyme A are essential nutrients and coenzymes in the human body. A lack of pantothenic acid and coenzyme A can lead to DNA loss and mutation, thereby increasing the risk of developing tumors [36]. In mouse models, studies have shown that pantothenic acid and its metabolite coenzyme A help CD8 + cytotoxic T cells differentiate into Tc22 cells that produce interleukin 22 and thereby enhance the antitumor functions of the body [37]. Therefore, we speculate that a similar mechanism is involved in the occurrence and development of ESCC; imbalances in the gut microbiota significantly increase the abundance of Gammaproteobacteria, Enterobacteriaceae, Lactobacillus, and other species belonging to Proteobacteria and Pseudomonadota and disrupts the balance of intestinal metabolites. Inhibition of the pantothenate and coenzyme A biosynthesis pathways significantly reduced the contents of pantothenic acid and L-aspartic acid and weakened the antitumor function of the body (Fig. 5A), which affects the occurrence and development of ESCC.

Research has revealed an interaction between tumor cells and inflammatory cells in ESCC, and inflammation-related parameters can serve as biomarkers for the prognosis of ESCC [38]. Therefore, we measured the WBC, neutrophil, and basophil levels in the blood of ESCC patients, which can indirectly reflect the inflammatory response and immune status of the body. Correlation analysis revealed that the number of peripheral blood cells in ESCC patients may be affected by the gut microbiota and intestinal metabolites. These patients showed a significantly increased abundance of Streptococcus in the gut microbiota and significant increases in the levels of the intestinal metabolites sphingosine and tetrahydrocortisone, leading to a reduction in eosinophils in the peripheral blood. Eosinophils participate in a variety of homeostatic processes, including metabolism, microbiome regulation, and immune regulation. In addition, eosinophils secrete a variety of cytokines, including eosinophil cationic proteins, neurotoxins from eosinophils, peroxidases and major basic proteins, that exert antitumor effects or promote tumor progression. Eosinophil infiltration is related to favorable prognoses in colorectal cancer, breast cancer and prostate cancer patients [39]. In addition, we found that the content of basophilic granules gradually increased during ESCC progression, and the increase in the number of basophilic granules may contribute to the proliferation and metastasis of ESCC. Research shows that basophils infiltrate various human cancers and induce immune protection in the tumor microenvironment by secreting cytokines such as IL-4/IL-13 to polarize macrophages to the M2 phenotype [40–42]. These results indicate that the abundance of pathogenic bacteria, such as Streptococcus and Shigella, in the intestines of ESCC patients is significantly increased and has an impact on monocytes and granulocytes in the peripheral blood, which imbalances the relationship between the systemic inflammatory response and immune response and may lead to the deterioration of ESCC; however, further research is needed to confirm this hypothesis (Fig. 5B).

Due to the lack of knowledge about whether gut microbiota is associated with the classification of esophageal squamous cell carcinoma in our study. We have only conducted preliminary explorations and have not controlled for gut microbes by immunohistotyping esophageal squamous epithelial cell carcinoma tissue, blood phenylalanine levels, or other metabolites in the blood. On the one hand, we want to combine experimental and clinical work to better guide clinical work, and on the other hand, we are limited by our technical level and research funding. The intestinal microorganisms may transfer from the intestine to the tumor lesions through the intestinal barrier, releasing cellular components that can shut down the immune system or cause inflammation, thereby promoting tumor growth. Whether the intestinal flora promotes the occurrence and development of esophageal squamous cell carcinoma through this pathway is currently unclear and requires further in-depth exploration. Of course, this is an interesting research direction, which may help us to better understand the relationship between gut microbiota and tumor development. In future studies, we will consider these factors and conduct more comprehensive experimental design to better explain the interaction between gut microbiota and tumor development.

In addition to simply describing the changes in the intestinal microbiota and metabolites in ESCC patients, we generated receiver operating characteristic (ROC) curves to identify potential intestinal biomarkers for the early diagnosis and prediction of ESCC. In our study, we found that the abundance of Fusobacteriaceae and Lactobacillus in the intestines of ESCC patients increased significantly, the content of the metabolite gibberellin A34 increased significantly, and the content of 12-hydroxydodecanoic acid decreased significantly. The areas under the receiver operating characteristic curve (AUCs) obtained using the levels of these markers were 81.44%, 86.04%, 82.56%, and 85.33%, respectively, which indicates that these markers are potential diagnostic markers of ESCC. Fusobacterium Fusobacteriaceae, which is closely related to the occurrence and development of ESCC, can invade and survive in aging ESCC cells, promote DNA damage, and further activate the DNA damage response pathway, thereby enhancing the aging-related secretory phenotype through paracrine signaling and promoting ESCC recurrence and chemotherapy resistance [16]. Increases in the expression of interleukin-32/protease-3 (PRTN3) and activation of the PI3K/AKT signaling pathway promote ESCC proliferation [17]. A study of the esophageal flora in ESCC patients revealed that lactobacilli are significantly enriched in esophageal tumor tissue. Lactobacilli in tumor tissue may affect the survival and prognosis of ESCC patients by influencing immune infiltration in the tumor microenvironment [22, 43, 44]. However, no relevant research has investigated the relationship between gibberellin A34 or 12-hydroxydodecanoic acid and ESCC, and additional research is needed to further explore this relationship. Nevertheless, these diverse gut microbes and metabolites can be used to distinguish ESCC patients from healthy individuals and have diagnostic value for ESCC.

## Conclusion

Our study preliminarily revealed changes in the gut microbiota and intestinal metabolites in ESCC patients. The gut microbiota of these patients exhibited an imbalance; the abundances of Shigella, Streptococcus and other pathogenic bacteria significantly increased; the abundances of beneficial bacteria such as Roseburia, Faecalibacterium and Veillonellaceae significantly decreased; the balance of intestinal metabolites was altered; and amino acid metabolism was significantly disrupted. In addition, ESCC patients exhibited significant increases in the abundance of Fusobacteriaceae and Lactobacillus in the intestine and in the abundance of the metabolite gibberellin A34 and significant decreases in the level of 12-hydroxydodecanoic acid, suggesting that these metabolites are potential diagnostic predictive markers for ESCC. A significant increase in the abundance of bacterial genera such as Enterobacteriaceae and Lactobacillus can lead to significant decreases in the contents of L-aspartic acid, pantothenic acid, and other products. By inhibiting pathways such as pantothenate and CoA biosynthesis, these products may be involved in the occurrence and development of ESCC. In addition, the observed imbalance in the gut microbiota may also cause a decrease in eosinophils in the peripheral blood, which may lead to inflammatory reactions and immune dysfunction in the body and thus ESCC deterioration. In conclusion, our research provides a large amount of evidence about the role of the gut microbiota in the pathogenesis of ESCC and provides new ideas for the early diagnosis, individualized treatment and targeted intervention in ESCC patients. This study may have potential bias; our sample size was small, and some differences were not significant. However, the sample size will be further increased in the future, and additional results need to be verified through further animal and cell experiments.

### Electronic supplementary material

Below is the link to the electronic supplementary material.

Supplementary Material 1

## Data Availability

All the raw sequences were deposited in the NCBI Sequence Read Archive under accession number PRJNA997054.
