# Supplementary material for: Mechanism of intestinal microbiota disturbance promoting the occurrence and development of esophageal squamous cell carcinoma——based on microbiomics and metabolomics
Source: BMC Cancer. 2024 Feb 22;24:245. doi: 10.1186/s12885-024-11982-8 (PMC10885407; doi:10.1186/s12885-024-11982-8)
Supplement: Supplementary file 1 — Supplementary Material 1 [file 12885_2024_11982_MOESM1_ESM.docx]

Supplementary Figure: Differential metabolite composition





Z score (standard score) graph: the abscissa is the value of the relative content of metabolites in the group converted by the Z score. The more to the right it is, the more metabolites there are in the group. The vertical axis shows the metabolite names.

Schedule S1: Relationship between metabolites and metabolic pathways of the ESCC group

| Number | ID | Enrichment of metabolic pathways in the ESCC group | Enrichment of metabolites  in the ESCC group | Impact |
| --- | --- | --- | --- | --- |
| 1 | hsa04210 | Apoptosis↑ | Sphingosine↑ | 0.2500 |
| 2 | hsa00140 | Steroid hormone biosynthesis↑ | Estrone↑  Cortisol↓  18-Hydroxycorticosterone↑  2-Hydroxyestrone↑  Tetrahydrocortisone↑  Cholesterol sulfate↑ | 0.0809 |
| 3 | hsa04977 | Vitamin digestion and absorption↑ | Ascorbate↑  Riboflavin↑  Pantothenic acid↓ | 0.0612 |
| 4 | hsa04211 | Longevity regulating pathway↑ | 1,1-Dimethylbiguanide↑ | 0.1250 |
| 5 | hsa04217 | Necroptosis↑ | Sphingosine↑ | 0.0833 |
| 6 | hsa04917 | Prolactin signaling pathway↑ | Estrone↑ | 0.0526 |
| 7 | hsa04921 | Oxytocin signaling pathway↑ | Prostaglandin H2↑ | 0.0833 |
| 8 | hsa04976 | Bile secretion↓↑ | Spermidine↓  Uric acid↓  Bilirubin↑  Cortisol↓  Lithocholic acid↑ | 0.0550 |
| 9 | hsa00250 | Alanine, aspartate and glutamate metabolism↓↑ | L-Aspartic acid↓  2-Keto-glutaramic acid↑ | 0.1790 |
| 10 | hsa00410 | beta-Alanine metabolism↓ | L-Aspartic acid↓  Spermidine↓  Pantothenic acid↓ | 0.1552 |
| 11 | hsa00360 | Phenylalanine metabolism↓ | Phenylethylamine↓  Phenyllactate↓  N-Acetyl-L-phenylalanine↓ | 0.1098 |
| 12 | hsa00770 | Pantothenate and CoA biosynthesis↓ | L-Aspartic acid↓  Pantothenic acid↓  D-4'-Phosphopantothenate↓ | 0.1518 |
| 13 | hsa00240 | Pyrimidine metabolism↓ | Methylmalonic acid↓  Thymine↓  dCMP↓  Cytidine↓ | 0.0508 |
| 14 | hsa00072 | Synthesis and degradation of ketone bodies↓ | (R)-3-Hydroxybutyric acid↓ | 0.1364 |
| 15 | hsa04960 | Aldosterone-regulated sodium reabsorption↓ | Cortisol↓ | 0.2000 |
| 16 | hsa00270 | Cysteine and methionine metabolism↓ | S-Adenosylmethionine↓  L-Aspartic acid↓  5'-Methylthioadenosine↓  L-Methionine S-oxide↓ | 0.0418 |
| 17 | hsa05133 | Pertussis↓ | Nicotinic acid↓ | 0.1000 |
| 18 | hsa04122 | Sulfur relay system↓ | S-Adenosylmethionine↓ | 0.0667 |
| 19 | hsa05215 | Prostate cancer↓ | Cortisol↓ | 0.0769 |
| 20 | hsa04927 | Cortisol synthesis and secretion）↓ | Cortisol↓ | 0.0556 |

Schedule S2: Blood indicators of ESCC patients at different tumor stages

| Name | T1 (n=6) | T2 (n=6) | T3 (n=12) | T4 (n=6) | Unit | Reference range | F | *P* |
| --- | --- | --- | --- | --- | --- | --- | --- | --- |
| WBC | 5.300±1.205 | 6.200±1.847 | 5.908±1.483 | 5.843±1.469 | 10^9^/L | 3.50~9.50 | 0.376 | 0.771 |
| RBC | 4.597±0.474 | 4.723±0.553 | 4.452±0.464 | 4.415±0.634 | 10^12^/L | 3.80~5.10 | 0.687 | 0.568 |
| HGB | 148.35±17.394 | 147.833±18.841 | 143.633±11.018 | 145.917±15.735 | g/L | 115.00~150.00 | 0.178 | 0.911 |
| PLT | 190.533±55.536 | 216.333±38.967 | 201.950±40.029 | 211.067±60.313 | 10^9^/L | 125.00~350.00 | 0.346 | 0.792 |
| NEU | 2.783±1.693 | 4.113±1.840 | 3.836±1.269 | 3.889±1.090 | 10^9^/L | 1.80~6.30 | 1.026 | 0.397 |
| LYM | 1.522±0.677 | 1.583±0.727 | 1.621±0.694 | 1.533±0.718 | 10^9^/L | 1.10~3.20 | 0.036 | 0.991 |
| MON | 0.315±0.085 | 0.315±0.109 | 0.324±0.110 | 0.360±0.102 | 10^9^/L | 0.10~0.60 | 0.250 | 0.861 |
| ESO | 0.085±0.029 | 0.152±0.164 | 0.112±0.098 | 0.102±0.085 | 10^9^/L | 0.02~0.52 | 0.005 | 0.722 |
| BAS | 0.019±0.009 | 0.020±0.009 | 0.023±0.016 | 0.035±0.016 | 10^9^/L | 0.00~0.060 | 2.861 | 0.056 |
| ALB | 46.933±10.940 | 41.217±4.855 | 40.525±3.513 | 41.217±4.900 | g/L | 40.00~55.00 | 1.599 | 0.214 |
